# What Is Considered Healthy Eating? An Exploratory Study among College Students of Nutrition and Food Science

**DOI:** 10.3390/nu16091365

**Published:** 2024-04-30

**Authors:** Maria Clara de Moraes Prata Gaspar, Claudia Soar, Mari Aguilera, Maria Clara Gomez, Ricard Celorio-Sardà, Oriol Comas-Basté, M. Carmen Vidal-Carou

**Affiliations:** 1 Departament d’Antropologia Social, Facultat de Geografia i Història, Universitat de Barcelona (UB), Carrer de Montalegre 6, 08001 Barcelona, Spain; demoraesprata@ub.edu (M.C.d.M.P.G.); mariaclarag.nut@gmail.com (M.C.G.); 2 Institut de Recerca en Nutrició i Seguretat Alimentària (INSA-UB), Universitat de Barcelona (UB), Av. Prat de la Riba 171, 08921 Santa Coloma de Gramenet, Spain; r.celorioisarda@ub.edu; 3 Nutrition Post-Graduate Program, Department of Nutrition, Federal University of Santa Catarina, Florianopolis 88040-900, Brazil; claudiasoar@hotmail.com; 4 Departament de Cognició, Desenvolupament i Psicologia de l’Educació, Secció Cognició, Facultat de Psicologia, Universitat de Barcelona (UB), Passeig de la Vall d’Hebron 171, 08035 Barcelona, Spain; mari.aguilera@ub.edu; 5 Institut de Neurociències (UBNeuro), Universitat de Barcelona (UB), Passeig de la Vall d’Hebron, 171, 08035 Barcelona, Spain; 6 NeuroDevelop eHealth Lab, eHealth Center, Universitat Oberta de Catalunya (UOC), Rambla de Poble Nou 156, 08018 Barcelona, Spain; 7 Departament de Nutrició, Ciències de l’Alimentació i Gastronomia, Campus de l’Alimentació de Torribera, Universitat de Barcelona (UB), Av. Prat de la Riba 171, 08921 Santa Coloma de Gramenet, Spain

**Keywords:** healthy eating, perception, college students, dietetics, nutrition, food technology

## Abstract

In modern industrialized societies, the focus on healthy eating has increased significantly across multiple sectors, including the media, public policy, expert opinion, and public awareness. The aim of this research was to explore the perceptions of healthy eating and the barriers to adopting a healthy diet among undergraduate students in Human Nutrition and Dietetics (HND) and Food Science and Technology (FST) degrees in Spain. An exploratory and descriptive cross-sectional study was conducted using a qualitative and quantitative methodology and convenience sampling. Two focus groups and a questionnaire were utilized (300 participants from all academic years completed the survey). Differences in definitions of healthy eating and perceived barriers were found between genders and students at different stages of training (*p* < 0.05). In their understanding of healthy eating, the students placed importance on balance, variety, moderation, and individual factors. Although students considered it easy to follow a healthy diet, family’s eating habits, time availability, and emotional states were found to be the main barriers to the implementation of healthy practices. The obtained data supports the need to critically address perceptions of healthy eating throughout the training of nutrition and food science professionals. The insights obtained on the perceived barriers highlight the importance of considering both individual and environmental factors.

## 1. Introduction

In industrialized societies, the issue of healthy eating has become omnipresent in discourses of the media, public policy, and expert opinion, and is a subject of increased public awareness [1]. The growing concern about the effects of diet on health is due to changes in the epidemiological, demographic, and nutritional profiles of the population [2,3]. Since 1990, the prevalence of obesity among the adult population has more than doubled worldwide, reaching 16% of the adult population in 2022 [4]. At the same time, nowadays, cardiovascular diseases are the main cause of death globally, representing 32% of all global deaths [5]. Parallel and paradoxically, thinness has become progressively valued and fatphobia, the discrimination and stigmatization against fat individuals, has become a phenomenon widely present worldwide [6,7,8]. Finally, food crises with important health, social and economic international impacts, such as the “mad cow”, have also increased concerns about food consumption [9].

Attempts to define what constitutes a healthy diet and provide dietary recommendations have been made in different scientific fields and organizations, including the World Health Organization (WHO). In response to the increase in prevalence rates of morbidity and mortality associated with chronic non-communicable diseases, in 2004 the WHO approved the Global Strategy on Diet, Physical Activity and Health, which invites member states to develop public policies to promote healthy eating and lifestyles [10]. To support and complement national initiatives, the European Union has also developed a common framework for action through the Green Paper, “Promoting healthy diets and physical activity: a European dimension for the prevention of overweight, obesity and chronic diseases” [11], and the White Paper, “A strategy for Europe on nutrition, overweight and obesity-related health issues” [12]. Several countries have developed public health policies along the same line, including the Spanish Strategy for Nutrition, Physical Activity, and Prevention of Obesity (NAOS Strategy) [13].

A universal science-based nutritional definition of a healthy diet is lacking [14], with the concept remains ambivalent and polysemous [15]. Studies show that the notion of “healthy eating”, from both the layman and professional point of view, does not encompass a single set of norms but can be understood in different ways depending on gender, age, social class, and cultural origin [15,16,17,18]. Furthermore, this complexity is increasing with the emergence of new forms of consumption, reorganization of the food chain, new agri-food production models, and the use of biotechnology [19]. Notably, as it evolves, the notion of healthy eating is being progressively associated with sustainability [20,21,22].

Professionals in the fields of health and food, such as dietitians, food scientists and technologists, play a central role in promoting healthy eating, as well as in the transmission of nutritional recommendations. These and other professionals working in healthcare fields may also have diverse perceptions of what constitutes healthy eating [15,18]. Understanding their conceptions of this notion is therefore essential, including during the formative period at university, as the insights gained may allow academic programs to be adjusted and improved if necessary [23,24]. A study carried out with Canadian dietetics students found that “healthy eating” was often associated with the inclusion of all groups of the food pyramid, moderation, balance, and individual customization. The students also felt that to be healthy, eating should be a pleasant experience and make you feel good about yourself [25]. A study conducted in Brazil verified that although nutrition students defined the key factors of healthy eating in accordance with national guidelines, they expressed a diversity of attitudes that needed addressing during their training [23].

Considering that the perception of what is “healthy” among professionals in the field of food and health can influence their personal and professional practices, it is essential to understand how they perceive this notion. To the best of our knowledge, such an analysis has not been carried out in Spain with university students of degrees in nutrition and food science. Therefore, this study sought to understand the perceptions of healthy eating and the barriers to its achievement among students from Human Nutrition and Dietetics (HND) and Food Science and Technology (FST) degrees at a Spanish public university.

## 2. Materials and Methods

### 2.1. Study Design

An exploratory and descriptive cross-sectional study aiming to comprehend the perceptions of food and healthy diet among college students was conducted between May 2020 and September 2021 by an interdisciplinary team using both qualitative and quantitative methodologies.

### 2.2. Setting and Sample

The research was carried out with a convenience sample of male and female college students enrolled in any of the four years of the bachelor’s degrees in HND and FST at the University of Barcelona (UB), an important institution in these fields in Spain. As defined by the UB, the HND degree trains professionals to carry out a range of health-related activities, including the promotion of adequate nutrition according to physiological or pathological needs, and a dietary and nutritional approach to the treatment and prevention of disease. The FST degree is aimed to train future professionals with the expertise to design and implement the best methods of producing, packaging and conserving foods, conducting research into new formulations and technologies to comply with the current demands for quality, safety and sustainability.

University students were chosen as the study sample considering that the university stage is fundamental in the life of an individual and involves various transformations, making healthy eating a challenge [26]. Therefore, promoting a healthy eating pattern and an appropriate relationship with food is essential at this stage of the life cycle and, to achieve this, it is crucial to know food perceptions. Moreover, nutrition and food sciences college students were targeted precisely because of the role that these students will have in food systems in their future professional lives. If their perceptions and knowledge concerning food and healthy eating influence their recommendations, it is essential to know and analyze them.

No exclusion criteria were defined regarding the age of the students, place of residence, and nationality. To the characterization of the sample, data were collected about educational level, course year, gender, age, municipality of residence, type of cohabitation, work activity or internship, average monthly household income, and parental level of education. The study followed the principles of the Declaration of Helsinki and was approved by the Bioethics Commission of the UB, protocol number IRBOC003099 (2020).

### 2.3. Data Production and Analysis

A questionnaire was created specifically for the research [24] based on data obtained in previous work on the food perceptions of HND college students and/or dietitians [15,23,27] and the general population [16,28,29], as well as based on the discourses obtained in the qualitative phase of the study.

The qualitative phase was carried out through focus groups, a useful method in the exploratory stages of a study, involving group discussions on a specific theme introduced by the researcher [30]. The collected data allows us to understand the construction of social perceptions and to refine research questions [31]. Two focus groups were organized with 13 students (11 females): five from HND and eight from FST. Participants were recruited through an email sent to all students enrolled in the degrees, and those who answered positively took part in the focus groups. According to the restrictions imposed by the COVID-19 pandemic, the two focus groups of October/November 2020 were conducted online via the Zoom platform. All participants had a microphone and video camera.

The focus groups, lasting 1 h and 30 min, were led by a researcher specialized in socio-anthropology of food and qualitative techniques, and observed by a master’s degree student in anthropology. The focus groups were guided by an interview script elaborated for the study, composed of open questions related to food perceptions and practices. The questions were later incorporated into the questionnaire used in the quantitative phase to improve its accuracy and as a complementary part of the data. The focus groups were recorded with participant consent and transcribed verbatim.

The thematic analysis of the discourses was carried out [32] by coding the content in analytical categories defined according to the objectives of the study and the emerging themes: perceptions of healthy food and eating, perceptions of food, trust/distrust of food, food sustainability, culinary activity, food choices, vegetarianism, and changes in perception during the degree studies. Initially, two researchers independently read the transcripts to identify the categories. They then compared this initial analysis and determined the final categories by discussion. Finally, the coding and systematization of the discourses was conducted using the software Atlas-Ti (version 8, Visual Qualitative Data Analysis, 2017).

Based on the analysis obtained though the qualitative phase, a questionnaire was designed and sent for review to 20 experts from different fields: nutrition (12), statistics (1), anthropology (5) and sociology of food (2). Adjustments were made based on their comments regarding question clarity, significance, and pertinence. A pilot test was conducted with 30 students, who were invited to comment on the questionnaire after completing it. The final questionnaire was composed of 31 multiple choice or Likert scale questions (including those for the sample characterization) relating perceptions related to food, healthy eating, and sustainability issues. Furthermore, an open-ended question was proposed: “Which word do you associate with the concept of “food”? (Indicate one word only)”.

The questionnaire was administered online between April and May 2021 through the Survey Monkey website. A total of 385 responses were obtained, 85 of which were excluded because they were incomplete, leaving 300 complete responses (78.0%). Out of the 300 participants, 151 were studying HND and 149 FST, representing 47.0% and 45.0% of the total enrolments in the two degrees, respectively. The overall mean age was 21.25 (±3.16) years, 21.76 (±3.80) for HND students and 20.73 (±2.24) years for FST students. Most of the informants were female (80.3%) (χ2 = 11.53, *p* = 0.030). Additionally, 81% of the sample presented a healthy Body Mass Index (BMI) (18.50–24.99 Kg/m^2^). More details regarding the sample characteristics are available in Gaspar et al. (2023) [24].

For the analysis, second and third year students were grouped together, resulting in three groups (first, second/third, and fourth year students). All data derived from the questionnaire were entered into SPSS v. 24 for statistical analysis. Descriptive results were expressed as means and standard deviations or frequencies, according to the nature of the data. Statistical results were obtained by comparing the distribution of response frequencies using the chi-square statistic, which was considered significant when *p* < 0.05.

## 3. Results

### 3.1. Perceptions of Own Health, Body, and Diet

**Figure 1:**
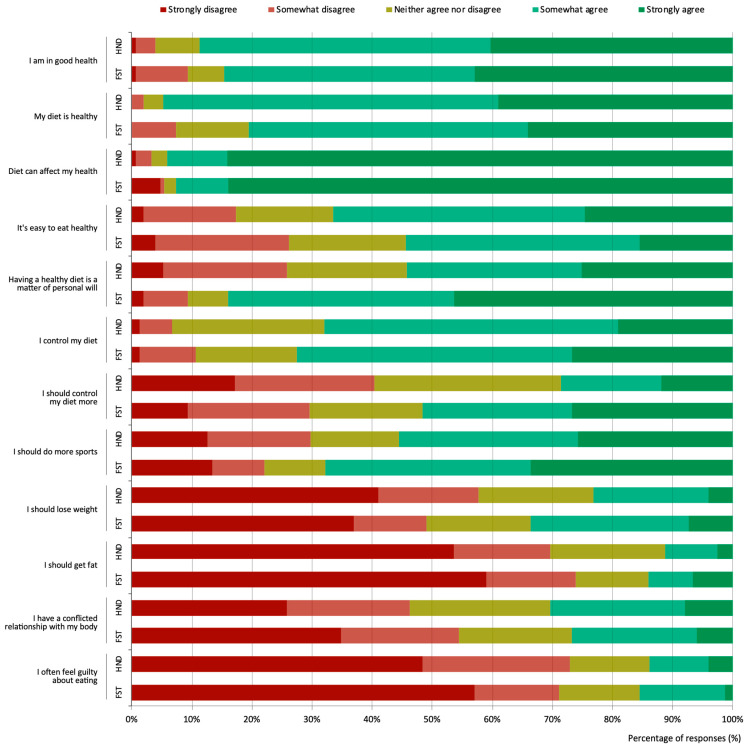
Perceptions of own health, body, and diet according to Human Nutrition and Dietetics (HND) and Food Science and Technology (FST) degree students.

The majority of both HND (88.7%) and FST (84.6%) students somewhat or strongly agreed that they were in good health (less than 1% strongly disagreed) (Figure 1). There was also a generalized perception that food consumption affects health, with no difference between degrees (84.1% of HND and 83.9% of FST students strongly agreed with this idea). Most informants considered their diet to be healthy, although the tendency was higher in HND students (χ2 = 16.16, *p* = 0.003): 91.0% somewhat or strongly agreed that their diet was healthy, compared to 80.4% of FST students. Likewise, more HND (66.2%) than FST students (54.3%) somewhat or strongly agreed that following a healthy diet is easy. Conversely, far more FST informants somewhat or strongly agreed with the item “having a healthy diet is a matter of personal will”: 83.9% versus 54.4% of HND students (χ2 = 32.20, *p* < 0.001) (Figure 1).

No significant differences were found between the proportion of HND (67.9%) and FST (72.4%) students who somewhat or strongly agreed that they controlled their diets, but significantly more FST students (51.6%) believed they needed more control over their diets compared to HND students (28.5%) (χ2 = 19.45, *p* < 0.001). Likewise, 67.8% of FST and 55% of HND students felt they should engage in more sports, with moderate or strong agreement (Figure 1).

Furthermore, 23.2% and 33.6% of HND and FST students, respectively, somewhat or strongly agreed that they should lose weight, whereas only 11.2% of HND and 14.1% of FST students felt they should gain weight, without significant differences between degrees in either case. Nearly 30% of participants from both degrees somewhat or strongly agreed that they have a conflictive relationship with their body, with 13.9% of HND and 15.4% of FST students stating that they feel guilty when eating (Figure 1).

Among HND students, the perception that diet can affect health was found to increase with the level of training, 83.0% agreeing in the first year compared to 97.7% in the fourth year (χ2 = 17.04, *p* = 0.030). Additionally, agreement with the idea that following a healthy diet is a matter of personal will decreased from 76.6% in the first year to 27.9% in the fourth year (χ2 = 30.22, *p* < 0.001). Among FST students, fewer agreed that “they should gain weight” at the beginning of the degree than in subsequent years (8.1% in the first year, 20.4% in the second/third year, and 11.9% in the fourth year) (χ2 = 17.06, *p* = 0.002).

Compared to men, more women believed that they should engage in more sports (63.1% versus 55.1%) (χ2 = 10.15, *p* = 0.003), had a conflictive relationship with their body (31.5% versus 15.5%) (χ2 = 11.16, *p* = 0.002), and felt guilty more frequently when eating (16.2% versus 6.8%) (χ2 = 11.07, *p* = 0.002). In contrast, more men than women agreed with the idea that they needed to gain weight (25.8% versus 9.5%) (χ2 = 30.06, *p* < 0.001).

### 3.2. Perceptions of a Healthy Diet

In this analysis of perceptions of healthy eating, qualitative data are presented before the quantitative results.

**Figure 2:**
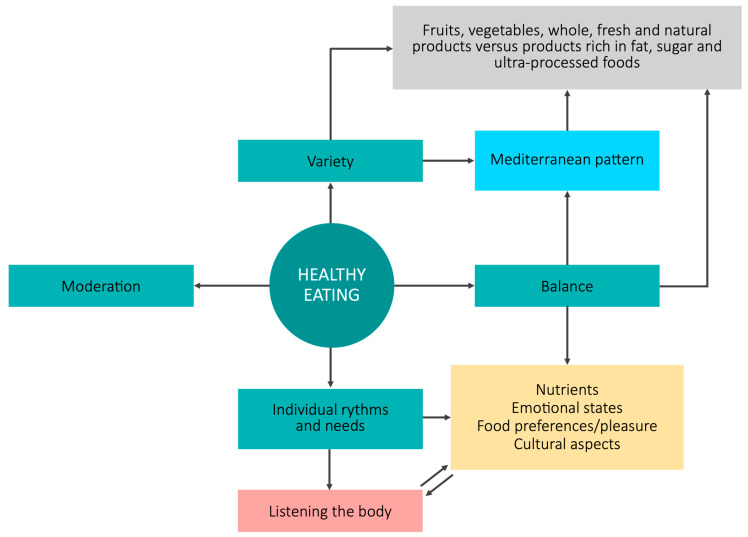
Main perceptions of a healthy diet of the according to qualitative data of the Human Nutrition and Dietetics (HND) and Food Science and Technology (FST) degree students.

The topic of healthy eating was the main subject of discussion in the focus groups, reflecting its importance for the students, who showed they had internalized certain general principles such as variety (“a variety of foods”, “a little of everything”), balance, and moderation (Figure 2 summarizes the qualitative results):

“*…a healthy diet is a balanced diet where you eat all kinds of foods, mainly healthy foods, but then if you eat something less healthy, as it’s a balanced diet, it’s OK, because your body can take it.*”

(Mireia, first-year HND)

“*Basically, I think it’s a varied diet. You can eat everything, but in the right amount, without going over the top.*”

(Alba, fourth-year FST)

They also associated healthy and balanced eating with a Mediterranean dietary pattern. Fruits, vegetables, and fresh, natural, and whole-grain products were perceived as healthy, whereas foods rich in fats and sugars, as well as processed products, were considered less healthy:

“*I’m trying to eat a lot of natural unprocessed foods. I basically concentrate on that, and that they don’t have much fat, you know bad fat, and things like that (…) because processed foods have, I don’t know how to put it, they have a lot of chemicals that aren’t good for my body*.”

(Mireia, first-year HND)

Likewise, for the participants, a balanced diet, considered a fundamental aspect of healthy eating, is one that provides adequate nutrients and goes beyond the mere calculation of ingested calories:

“*This thing of counting calories, I personally consider it to be overrated, that is, I don’t think that someone’s diet should be judged by the number of calories it has or doesn’t have but whether it is balanced. You have to know where you can get what you need. For example, I’m a vegetarian, so I need to know where protein comes from. I have to know where I’m going to get sugars and fats from, but it’s necessary to have a balance between all these macronutrients and micronutrients*.”

(Marta, first-year HND)

“*It has to be balanced in micro and macro nutrients, but I don’t know, it’s not as if there’s a standard definition*.”

(Elena, fourth-year FST)

Students also perceived that healthy eating depends on each individual and their life stage, activities, and nutritional requirements. Thus, healthy eating is personalized:

“*I think it depends a little on each person, doesn’t it? On the dietary, nutritional requirements of each person, and yes, there is a basis…yes, there is a basis in the nutritional sense, isn’t there? Well, requirements that keep us healthy at a physiological level and a biological level as well*.”

(Samanta, first-year FST)

“*What is healthy for some people may not be healthy for someone else because the physiological characteristics are different for each person, and everything is healthy, because the poison is in the dose. (…) The amount and the physiological conditions, because eating wouldn’t be the same for me as for a 95-year-old, or a 6-year-old boy or girl*.”

(Eric, fourth-year FST)

Furthermore, the discussions, especially in the case of HND students, revealed a conception that goes beyond compliance with nutritional recommendations. Personalization of diet was also conceived as a balance between the physiological needs of each person, their food preferences, and demands associated with emotional states. Healthy eating was also related to listening to the body, implying eating what the body and mind ask for:

“*…giving my body what it is asking for, or not the body, but the mind, because we often feel like ‘okay, now I’d like to eat something lighter, or something more filling because I need this extra energy boost’. I believe that this is healthy eating, with variety but paying attention to what the body is telling you. I also believe that our diet is greatly influenced by what we think, our emotions, what we are experiencing, and sometimes our body is saying, give me a salad or give me something lighter but we eat biscuits to satisfy an emptiness, an emotional problem, to satisfy not our biological needs, but mental and emotional. We eat things for this reason and not for our body, so it has two dimensions.*”

(Gemma, first-year HND)

In this case, the suitability of the diet would depend on individual factors, both external and internal, and not only be based on nutritional balance according to biomedical knowledge. Pleasure, as well as cultural, symbolic, and social aspects, were also perceived as important:

“*That it adapts to your needs, both physiological, symbolic, cultural … your needs and your tastes, right?*”

(Amanda, fourth-year HND)

Some participants, especially from the FST degree, did not share this “holistic” vision, as they considered that one cannot eat “only” what the body or mind asks for. In this case, they distinguished between what is healthy and what is pleasant, two aspects held to be incompatible:

“*If what is healthy is what my body is asking for, I would eat French fries (laughs)…or something like that…you know?*”

(Anna, first-year FST)

“*If as they say it’s an individual thing…okay, right now I can say ‘vegetables don’t make me happy, sugar makes me much happier, things with sugar’, well my diet wouldn’t be healthy. If I start eating what really makes me feel good, it makes me feel good if it makes me happy…*”

(Claudia, first-year FST)

The quantitative data corroborated part of the discourses emerging from the discussion groups. Overall, “a balanced diet” was considered the most important aspect of healthy eating, followed by “respect the physiological signals of hunger and satiety”, although a statistical difference between HND and FST students was found regarding the latter (Table 1). Among HND students, the third most chosen factor was “eat fresh and natural foods”, which was in fourth position among FST students. “Eat a variety of foods/a little of everything” was the third most important for FST students, with a statistical difference between the two degrees in this respect. The pleasure of eating was the fourth most chosen factor by HND students, whereas it was not considered important by FST students, a significant difference again being evident between the degrees (Table 1).

Perceptions of what constitutes healthy eating also differed among the students according to the level of training. Among HND students, the importance attributed to “respect the physiological signals of hunger and satiety”, “eat five servings of fruits and vegetables a day”, and “eat in the company of others” increased significantly over the years (Table 1). In contrast, the selection of “consume all macronutrients, vitamins and minerals” decreased significantly among more advanced students. Among FST students, those in the first year considered it was most important to “eat a variety of foods, a little of everything”, whereas “consume all macronutrients, vitamins and minerals” and “eat five servings of fruits and vegetables a day” acquired significantly more importance in later years.

The analysis revealed that female and male participants shared similar perceptions, although significant differences were found in the importance attributed to “respect the physiological signals of hunger and satiety”, “eat according to the food pyramid” and “consume all macronutrients, vitamins and minerals” (Table 1).

### 3.3. Perceptions of Barriers to Adopting a Healthy Diet

**Figure 3:**
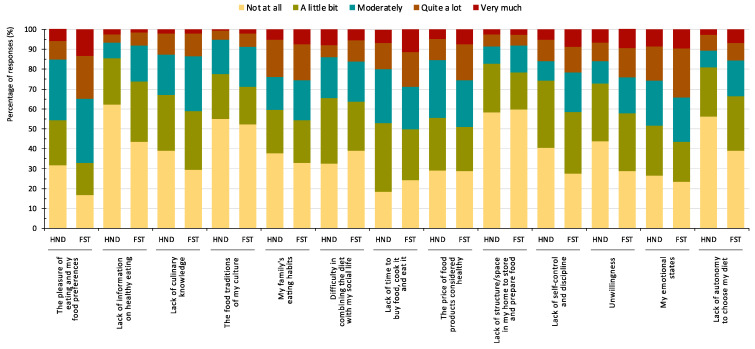
Barriers to healthy eating according to HND and FST degree students.

**Figure 4:**
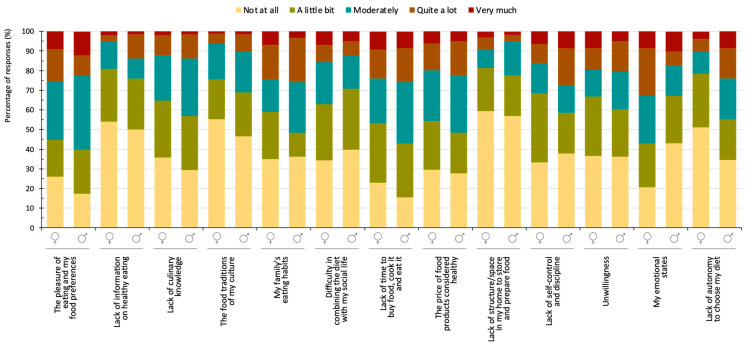
Barriers to healthy eating according to gender.

It is also crucial to understand perceived impediments to healthy eating (Figure 3 and Figure 4). Students of both degrees signaled three factors as the main barriers: “my family’s eating habits”, “lack of time to buy food, cook it, and eat it”, and “my emotional states”. “Lack of information about healthy eating” was judged to be of least importance, especially by HND students (χ2 = 13.96, *p* = 0.007) (Figure 3). “The pleasure of eating and my food preferences” was more influential for the FST group (χ2 = 20.21, *p* < 0.001), as was “lack of self-control and discipline” (χ2 = 10.61, *p* = 0.031), “lack of will” (χ2 = 9.56, *p* = 0.048) and “lack of autonomy to choose food” (χ2 = 12.80, *p* = 0.012).

Among the HND students, “lack of time to buy food, cook it, and eat it” became more important over time (χ2 = 15.99, *p* = 0.042), whereas “lack of information about healthy eating” had almost no influence on fourth-year students in contrast with those in the first year (χ2 = 61.11, *p* < 0.001) (Figure 3). For FST students, “lack of time to buy food, cook it, and eat it” (χ2 = 16.12, *p* = 0.041), “difficulty in combining a healthy diet with my social life” (χ2 = 22.97, *p* = 0.003), and “lack of structure/space in my home to preserve and prepare food” (χ2 = 21.40, *p* = 0.006) were significantly more important at the end of the degree than at the start.

Analysis by gender revealed only two statistical differences (Figure 4). Emotional states impeded women from following a healthy diet to a greater extent (χ2 = 17.68, *p* < 0.001): 32.8% of female students declared that this factor affected them a lot or very much, compared to only 17.2% of male students. Moreover, 67.2% of the men indicated that this factor affected them not at all or only a little compared to 43.1% of the women. “Lack of autonomy to choose my diet” influenced the male group significantly more (χ2 = 14.49, *p* = 0.009): 10.3% of women declared that this dimension was quite or very important compared to 24.1% of men. Also, 78.4% of women stated that this aspect did not influence them at all or only a little versus 67.2% of men.

## 4. Discussion

### 4.1. Perceptions of Own Health, Body, and Diet

To the best of our knowledge, this is the first study to analyze perceptions of healthy eating among HND and FST university degree students in Spain. To implement strategies that promote healthy eating and are adapted to the social context, it is essential to understand how individuals understand this concept and what meanings they attach to it [33]. In the case of professionals in the fields of health and food, insights into their conceptions of what constitutes “healthy” can indicate how these actors will carry out their professional practice [15].

Almost all the participants believed their diet to be healthy and that diet can affect health, an outcome that may be related to the fact they were studying degrees in nutrition and food. This perception was statistically more important among HND than FST students and increased over the years, which supports the observation that training in nutrition increases concern about diet and its relationship to health [34]. However, a similar perception was previously observed in the general university community (of the same institution), including students of other subjects, professors, and administrative staff, who also believed their diet to be healthy [22]. Likewise, most of the European population, especially women, consider that they usually follow a healthy and sustainable diet. Spain is among the top four European countries in which individuals have a positive view of their eating habits [35]. This can be a challenge to change food practices, since if individuals are convinced that they already eat well, they would be less sensitive to make changes.

The students’ positive assessment of their diet as healthy may also be associated with the growing medicalization of food over the last three decades. The tendency to relate food with health has influenced not only professionals in the field of health and nutrition (as mentioned above), but also the general population [36,37]. According to the Food Safety Barometer in Catalonia, concern that health is affected by diet is high among the Catalan public and the nutritional composition of food is the most influential factor at the moment of purchase [38]. Likewise, this emerged as the predominant reason for the dietary decisions of the students participating in the present work [24].

Nearly 85% of FST and 55% of HND students believed that “having a healthy diet is a matter of personal will”. Additionally, a high percentage of students in both degrees somewhat or strongly agreed that they controlled their diet and about half of the FST students somewhat or strongly agreed that they should control their diet more. These data indicate that, in general, the informants had internalized an individual responsibility regarding their diet. The process of medicalization has promoted a rational, nutritional, functional and reductionist conception of food, as well as a more preventive and individualized relationship, in which self-control and discipline are fundamental [36,37].

The sense of individual responsibility may also be related to the pressure of body normativity related to the hegemonic aesthetic models in Western industrialized countries, which are structured around fatphobia and valorization of thinness [6,39,40]. Although these models have been questioned (for example, through anti-fatphobia movements [41,42]), they still have an impact on the population, mainly on women, the young, and LGTBIQ+ groups [43,44,45], influencing eating practices and promoting slimming and/or restrictive diets, as well as other body-shaping methods [6,46,47]. HND students are generally reported to show a high rate of eating disorders [48,49,50]. It is notable that in the present study, more than half of the students in both degrees, especially women, somewhat or strongly agreed that they should engage in more sports. About a third of the FST students and a quarter of the HND students felt they needed to lose weight, about a third from both degrees had a conflictive relationship with their body, and about 15% felt guilty when eating. Women were also observed to be more concerned than men about losing weight in a recent study by Fayet et al. (2022) [47], who reported that female university students consider themselves more overweight than they really are, and in most cases feel obliged to diet or exercise to control or lose weight.

Discourses about food in Western societies therefore favor self-control and the act of eating is frequently associated with a bodily experience that requires the continuous exercise of self-discipline to conform to social norms [51]. As Ascher (2005) suggests, in modernity, new social regulations have been put in place that play with reflexivity, as well as with the responsibility of individuals regarding the act of eating. Thus, society “provides” the individual with knowledge to rationally choose what they eat, and they are repeatedly told in various ways that eating is an act of will and that what they eat, as well as the future of their body, depends on a capacity for self-control [52].

Although the perception of HND students that having a healthy diet is a matter of personal will diminished over the years of training, it is important to question how these student attitudes can influence future professional activity. For example, in nutritional consultations with patients, discourses that value thinness, body control, and individual responsibility may be reproduced, thereby fomenting a sense of guilt [6] that would be counterproductive in a food and nutritional education process. These topics therefore require more attention from a critical point of view throughout the training.

### 4.2. Perceptions of a Healthy Diet

According to the qualitative analysis, the conceptions of healthy eating are polysemic, although certain factors stand out: variety (privileging plant foods and fresh and natural products), balance, moderation, and respect for individual rhythms and needs (not only nutritional). Most of these results were verified by the quantitative analysis, which found that students of both degrees predominantly cited balance and respect for individual physiological signals (with a statistical difference between degrees for the latter). Next in importance was variety, especially for FST students, and the consumption of fresh and natural foods, which was selected more by HND students. Furthermore, both the qualitative and quantitative results showed that pleasure was more important for the future dietitians than the food technicians.

Associating the concept of balance with health and nutrition is far from new, being expressed by Hippocrates, considered the founding father of medicine and nutrition [53]. The idea remains strongly related to health and nutrition, as reflected in the WHO’s definition of a healthy diet. Numerous studies have revealed that individuals from different sociocultural contexts, including in Spain, associate healthy eating with balance [54,55,56,57,58].

As the present study reflects, the notion of variety is also important in the definition of healthy eating [56,59]. Odela (2004) found a widely shared consensus among Spaniards that “eating well” means eating a balanced and varied diet, with a little of everything [55]. In a study of young university women and Spanish dietitians, it was also verified that “healthy eating” is above all associated with the ideas of “balance”, “moderation” and “variety” [60]. For the European adult population, a healthy and sustainable diet mainly involves eating a “variety of different foods and having a balanced diet” and “eating more fruits and vegetables” (both 58%) [35].

The importance given by the students to consuming fruits and vegetables and fresh and natural foods is worth highlighting. In contemporary industrialized society, concern is growing about the increase in diet-related diseases [2,61] and the considerable environmental impact of consuming products of animal origin [21]. In this context, products of plant origin, especially fruits and vegetables, as well as those with minimal processing are widely considered to be healthier (fresher and more natural) and more sustainable in institutional discourses, both international [21] and national [13]. These themes have been assimilated by the lay public in different countries [26,29], including Spain [20,62], and the university community [15,22]. A Eurobarometer found that freshness is the most important food concern among Spanish consumers [29]. More recent data indicate that “eating more fruits and vegetables” is the most important aspect for Europeans when defining a healthy diet [63]. In a study with a representative sample of the Spanish population, it was verified that the four most important determinants of a healthy diet were held to be eating fresh products, an adequate combination of all types of foods, a diet that contains certain components (vitamins, minerals, fibers, etc.), and a good-for-you diet (that makes you feel good) [20].

Although it was observed that students of both degrees place importance on individual rhythms and feelings, their general conceptions of healthy eating did not include many social or sensory aspects, such as eating with other people, having contact with food, cooking, and pleasure. A notable bias toward a more medicalized perception of food [36,37] was especially evident among the FST students, which could be related to the more technical nature of their training compared to HND students. Indeed, the analysis revealed that over the years, HND students expressed a more holistic and subjective perception of healthy eating, placing significantly more value on, for example, “respect the physiological signals of hunger and satiety” or “eat in the company of other people”. In contrast, the FST students placed growing importance on nutritional aspects as the course progressed.

Regarding gender differences, male participants showed a more medicalized perception of healthy eating than women, which contradicts observations in the general population. Overall, women have a higher tendency to establish a connection between food and health, to diet (often driven by aesthetic norms of thinness), and to exhibit a medicalized approach to food [57,64,65]. This gender difference, associated with the social construction of gender roles [66], is even reflected in the profile of HND students, the majority of whom are women [67,68]. A possible explanation for this result could be related to the profile of men enrolled in courses related to health sciences, who usually already have a prior interest in diets, as well as the body and sports. Kaur et al. (2019) observed that more than a third of male health science students were worried about their body image and imposed dietary restrictions to control their weight or body shape [69]. Griffiths et al. (2018) also identified a relationship between concern about body image and the presence of eating disorders in male students [70]. Those studying physical education were strongly motivated to achieve a muscular appearance and applied strategies to achieve this, such as increasing protein intake and using sports supplements.

### 4.3. Perceptions of Barriers to Adopting a Healthy Diet

Although about 55% of FST and 65% of HND students somewhat or strongly agreed that following a healthy diet is easy, about 50% of the sample recognized some barriers. Food consumption is influenced by social, economic, cultural, political, and environmental determinants [71,72] which, as Gracia-Arnaiz (2021) indicates, “explain, in part, why even with full knowledge of the nutritional recommendations about what and how much to eat, certain food practices seem far removed from the optimal diet” [73]. Accordingly, complying with dietary recommendations constitutes a significant challenge and the barriers to their compliance are frequently overlooked [72]. In fact, individuals are often considered to be free and rational in their food decisions, which disregards the complexity of the act of eating [39,71,73] and assumes that assimilating knowledge inevitably results in better practices [6,72]. However, there are disparities between internalized norms and the practices of individuals [33]. Beyond these norms exists a regime of feasibility, characterized by the possibilities that subjects encounter in their daily lives [65,74].

In the present study, “my family’s eating habits” was the most cited factor that prevented participants from following a healthy diet. From early childhood, the family plays an important role in the construction of the dietary model of individuals through the process of primary socialization, in which the ways of being, thinking and acting of the sociocultural group are internalized, including food culture [75]. The medicalized conception of food, based on a reductionist perspective focused on nutritional and functional aspects, has been accentuated to the detriment of cultural, symbolic, and hedonic dimensions [33,36,37]. Some traditional foods and ways of eating have therefore come to be seen as unhealthy and too “heavy” according to this nutritional model. A study of Spanish, Brazilian, and French dietitians found that the process of professional socialization and the acquiring of technical nutritional knowledge generated tensions in the family environment, due to a discrepancy between practices at home and what was considered nutritionally optimal [34].

Another barrier to healthy eating for the students was the “lack of time to buy food, cook it, and eat it”. Time availability has become a determining factor in eating practices, especially among the urban population who have long commutes between home and the place of work/study. In contemporary life, the transformation of the time and space of everyday eating implies new modalities of the act of eating, forcing individuals into a “do-it-yourself” approach to temporal-spatial ordering [33] and making it difficult to follow a healthy diet. This context favors, for example, the consumption of industrialized products, which are often cheaper as well as faster and more convenient to prepare and eat [72]. In fact, since the 1970s, there has been a trend in European countries to dedicate less time to domestic culinary activity and more time to eating outside of home, as well as an increase in the consumption of highly processed products [76,77]. Other studies carried out in the general population and with university students have also identified the importance of time availability [65,78]. Among university students from the Arab Emirates, lack of free time appears as one of the barriers to healthy eating, and is associated with stress, being experienced more commonly at the end of the semester, the period of exams and project deadlines [62]. In the present study, as in previous work [78], lack of time statistically affected students more at the end of their degree, which may be associated with increased academic demands, but also with the acquisition of new responsibilities such as internships, entry into the world of work, and/or emancipation from the family home.

The third most important issue preventing students from following a healthy diet was “my emotional state”, which was indicated more by women than men. This finding is in line with recent studies reporting that the emotional state and its regulation are key factors in the choice and quantity of food eaten (see the recent review by Frayn and Knäuper, 2022) [79]. Interestingly, the use of maladaptive emotional regulation strategies has been related to eating disorders such as anorexia and bulimia [80]. In a study with a Spanish university population, it was shown that the emotional state was one of the main barriers to healthy eating, together with other individual (e.g., budget, planning) and environmental factors (e.g., price of food, geographical accessibility) [81].

Lack of information about healthy eating was held to be the least important barrier, especially by HND students, which may be due to their training, as the significance of this factor decreased statistically from the first to the fourth year of the degree. Furthermore, this result could be expected considering the interest of the participants in food. Other studies similarly report that insufficient information is not the main reason individuals fail to follow a healthy diet, with other factors (social or economic, for example) playing a more fundamental role [35].

A comparison between the two degrees revealed that “the pleasure of eating and my food preferences” was selected more by the FST than the HND students as a perceived barrier, as was “lack of self-control and discipline” and “lack of personal will”. These differences could be related to the different conceptions of healthy eating associated with their respective degree subjects, a more multidimensional perception being observed in HND students, who had a higher tendency to integrate pleasure into the concept of healthy eating. Additionally, FST students were statistically more likely to agree that following a healthy diet is a matter of personal will. A possible explanation is that FST students are trained to ensure the quality and safety of food from a physical, chemical, and microbiological point of view. They may therefore be more inclined to think that healthy eating depends more on choosing safe, contaminant-free foods rather than nutritional balance and adherence to a healthy diet. Unlike HND students, who study food and nutrition from a more holistic health-promotion perspective, FST students may take a more technical and food production-oriented approach [82].

When comparing genders, “lack of autonomy in choosing my diet” was significantly more relevant for the male students. Some men within this group may have felt greater pressure to adhere to certain dietary patterns, which could heighten their perception of a lack of autonomy if they were unable to meet those expectations. Our finding also supports that of Entrena-Durán et al. (2021), who reported that a higher percentage of women believed they were eating healthily compared to men, which aligns with existing societal norms and gender roles [65]. Accordingly, as women have traditionally been responsible for acquiring and preparing food, they have greater experience and a deeper understanding of healthy eating. In contrast, men living outside the family home or without female partners exhibited more feeding problems, potentially due to their limited exposure to these practical experiences. This indicates a potential disparity in food education between genders. Beyond promoting gender equality, encouraging men’s active involvement in food-related tasks can help overcome their relative lack of training in this domain. By acquiring the necessary skills and knowledge, men can improve their eating habits and overall nutrition, bridging the gap identified in both studies. It is important to note that addressing this issue requires a broader societal approach that challenges traditional gender roles and promotes inclusivity in domestic responsibilities. Education campaigns can play a vital role in promoting equal involvement in food-related tasks and fostering a healthier eating culture for both men and women.

## 5. Conclusions

To our knowledge, this is the first study to analyze perceptions of healthy eating among university students in the field of food science in Spain. In general, the students considered their diet to be healthy, although significant differences emerged between degree courses and genders. An internalized individual responsibility in relation to food and the body was identified, possibly influenced by the pressure of aesthetic norms of thinness and the medicalization of eating. These findings highlight the importance of critically addressing such attitudes during student training, especially in the field of nutritional care, to avoid the perpetuation of discourses that promote thinness and individual responsibility for body shape.

In their perceptions of healthy eating, the students highlighted balance, variety, moderation, and respect for individual physiological signals. FST students gave more importance to variety and the consumption of fresh and natural foods, whereas HND students were more likely to cite balance and pleasure in eating. Although most students felt it was easy to follow a healthy diet, barriers to the implementation of healthy practices were also identified, such as the family’s eating habits, time availability, and emotional states. As students spend a considerable part of their daily lives in university spaces, it is essential that institutions promote favorable conditions for healthy eating and develop strategies adapted to this group, considering environmental barriers as well as individual factors. Furthermore, the results indicated that it is necessary to promote, both during the training of these future professionals and at the public health level, messages about healthy eating that address all its complexity (not only the nutritional aspects) and that consider that its real application is complex, potentially encountering multiple barriers.

Although this study has produced significant data, particularly in the context of Spain, it has certain methodological limitations. Firstly, the research was conducted exclusively with students from a single academic institution in the Barcelona area, in Spain. The generalizability of the research could be increased by including participants from other institutions and geographical contexts (both at national and international level). Additionally, the use of convenience sampling may have introduced bias, as participants can share similar profiles or interests pertaining to the subject matter. Furthermore, despite the anonymous nature of the study, observational studies based on questionnaires about food practices and perceptions may encounter a potential information bias because individuals can respond as they think they should answer. Finally, relying solely on two focus groups for the qualitative phase of the study limits the depth of the collected data, and employing semi-structured interviews could have yielded more comprehensive insights.

## Figures and Tables

**Table 1:** Perceptions of a healthy diet of the college students enrolled in bachelor’s degrees in Human Nutrition and Dietetics (HND) and Food Science and Technology (FST) according to the degree, year of training, and gender.

Aspect Related to Healthy Eating	HND	FST	X^2^	HND			X^2^	FST			X^2^	Gender		X^2^
	Total (%)	Total (%)		1st (%)	2nd/3rd (%)	4th (%)		1st (%)	2nd/3rd (%)	4th (%)		Female	Male	
Eat fresh and natural food	12.4	12.1	0.02	11.3	12	14	0.63	12.2	13	10.6	0.56	12.4	10.9	0.42
Take pleasure in eating	9.5	4.3	11.31 ***	11.3	8.7	8.5	1.04	2	6.2	4.1	3.79	6.8	7.5	0.12
Respect the physiological signals of hunger and satiety	19.2	14.1	7.05 **	14.2	22.4	20.2	6.81 *	11.6	14.1	17.1	2.50	18.1	10.9	8.72 **
Have contact with food and cooking	5.3	2.9	3.57	6.4	5.5	3.9	0.97	2.7	1.1	5.7	5.72	4.3	3.4	0.27
Eat according to the food pyramid	2.2	5.6	7.51 **	4.3	5.1	1.6	4.24	6.8	5.1	4.9	0.70	3.2	6.9	5.62 *
Eat a variety of foods, a little of everything	8.8	13.6	7.01 **	8.5	8.2	10.1	0.45	18.4	11.3	11.4	6.06 *	10.4	14.4	3.02
Preferentially eat organic food	0.7	1.3	1.07	1.4	0.5	0	2.152	2	0.6	1.6	1.46	1.1	0.6	0.41
Eat while sitting at the table without looking at the television, computer, or smartphone	1.5	0.9	0.81	1.4	1.6	1.6	0.026	0.7	1.7	0	2.51	1.4	0.6	0.78
Eat in the company of other people	4.6	3.6	0.70	1.4	7.1	4.7	6.45 *	6.1	3.4	0.8	5.94	4.7	1.7	3.44
Consume all macronutrients, vitamins, and minerals	5.3	6	0.26	6.4	7.7	0.8	8.57 *	1.4	7.3	9.8	10.55 **	4.4	10.9	12.54 ***
Have a balanced diet	20.3	23	2.22	21.3	18.6	21.7	1.17	23.8	23.2	22	0.33	21.9	20.7	0.25
Respect the times of the main meals and do not snack between meals	2.9	5.8	5.18 *	4.3	2.2	2.3	1.50	8.2	5.6	3.3	3.38	4.3	4.6	0.04
Eat five servings of fruit and vegetables a day	4.6	4.7	0.002	2.8	3.3	8.5	6.88 *	1.4	6.8	5.7	6.26 *	4.3	6.3	1.44
Follow a vegetarian diet or one with a low consumption of animal products	2.6	2	0.42	5	1.1	2.3	4.97	2.7	0.6	3.3	3.35	2.8	0.6	3.95

* *p* = <0.05; ** *p* = <0.01; *** *p* <0.001.

## Data Availability

The raw data supporting the conclusions of this article will be made available by the authors on request.
